# A scoping review of turmeric adulteration based on data from six continents

**DOI:** 10.1080/13880209.2025.2606229

**Published:** 2025-12-26

**Authors:** Stefan Gafner, Nilüfer Orhan, Çiğdem Kahraman, Mark Blumenthal

**Affiliations:** ^a^ American Botanical Council, Austin, TX, USA; ^b^ Department of Pharmacognosy, Faculty of Pharmacy, Hacettepe University, Ankara, Türkiye

**Keywords:** Adulteration, *Curcuma longa*, dietary/food supplement, spice, turmeric

## Abstract

**Context:**

Turmeric (*Curcuma longa*) is widely used as a spice and in dietary/food supplements and herbal medicines. Reports assessing the authenticity of commercial products have shown that the ingredient is subject to adulteration with, among others, artificial dyes, undeclared diluents, and synthetic curcumin.

**Objective:**

This scoping review summarizes published data on adulteration of turmeric products sold as spice and dietary or food supplements to estimate the prevalence of non-authentic turmeric on the market.

**Methods:**

This scoping review was based on a literature analysis from Google Scholar, PubMed, ScienceDirect, Scopus, and Web of Science databases, covering publications from 2000 to 2025. Article selection was performed according to PRISMA-ScR guidelines. After the initial search, specific countries were added to refine the search. Of the 375 publications retrieved, 347 were eliminated as duplicates or because they lacked information on turmeric adulteration, adulteration of commercial products, or did not provide the number of adulterated samples. An additional 19 papers were found searching the citations, or by using Google Search with the keywords “Curcuma longa”, “turmeric”, “government report”, and “adulteration”. One more report from the CVUA Stuttgart was found using the keywords “Kurkuma”, “Verfälschung”, and “Report”. In total, 48 papers were included in the review.

**Results:**

A total of 48 publications representing 2235 commercial turmeric samples were included in the study. The overall adulteration rate was 20.0%, with spice samples having a slightly lower percentage of adulterated samples (20.4%) than dietary and food supplements (22.0%).

**Conclusion:**

Adulteration of turmeric remains a concern on markets worldwide.

## Introduction

The roots and rhizomes of turmeric (*Curcuma longa* L., Zingiberaceae) have a long history of use in food and medicine. Documented use dates back at least to the Vedic period (1500 − 500 BCE). (Buch et al. 2012, Akhila and Gopi, 2020) Akhila and Gopi mention the listing of turmeric in the *Agni Purana*, an ancient Sanskrit text dating back to the 7^th^ – 11^th^ century CE, as a remedy for jaundice, wound healing, and hemorrhoids, among other conditions. (Akhila and Gopi, 2020) Modern research, including human clinical studies, has shown mild benefits of turmeric for many conditions, with studies focusing especially on turmeric’s effects as an anti-inflammatory agent and to lower blood sugar and blood lipids. (Zeng et al. 2022, Hidayat et al. 2025, Ferguson et al. 2021, Jafari et al. 2024)

India is the world’s largest producer of turmeric, providing about 80% of the global turmeric. In the 2023–2024 season, the export value of turmeric from India was indicated to be 18.7587 billion rupees, corresponding to approximately US $226.5 million, according to a report of the Government of India. (Kumar et al. 2025) In 2023, the main importing countries were the USA, followed by India, Germany, Malaysia, Morocco, and China (Tridge 2025a). Prices for turmeric can vary substantially according to Tridge, a company providing information on food items. Tridge reports wholesale prices for fresh turmeric ranging between $5.63/kg and $9.55 USD/kg in 2023, and from $5.44/kg to $10.99/kg in 2024 (Tridge 2025b).

**Figure 1.**
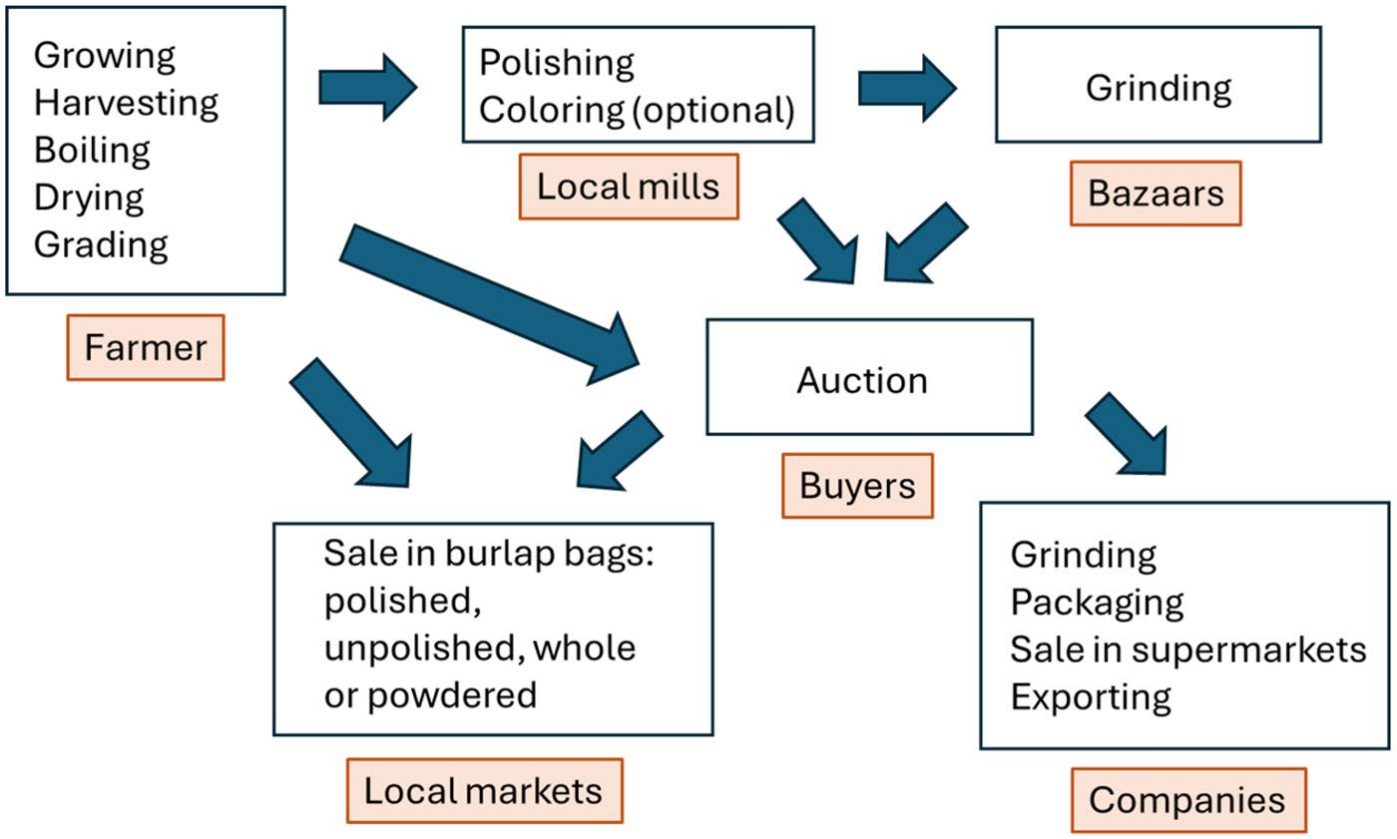
Turmeric supply chain for local use and smaller companies based on information from Forsyth et al. and Booker et al. (Forsyth et al. 2019a, Booker et al. 2014).

Growing turmeric on smallholder farms is still common in India and other producing countries. After the harvesting and drying, the turmeric roots and rhizomes are graded. Important criteria include color and appearance, aroma and flavor, moisture content, and visible contaminants. A typical turmeric spice supply chain for smaller manufacturers, based on information from Bangladesh and India, is shown in Figure 1.

**Figure 2.**
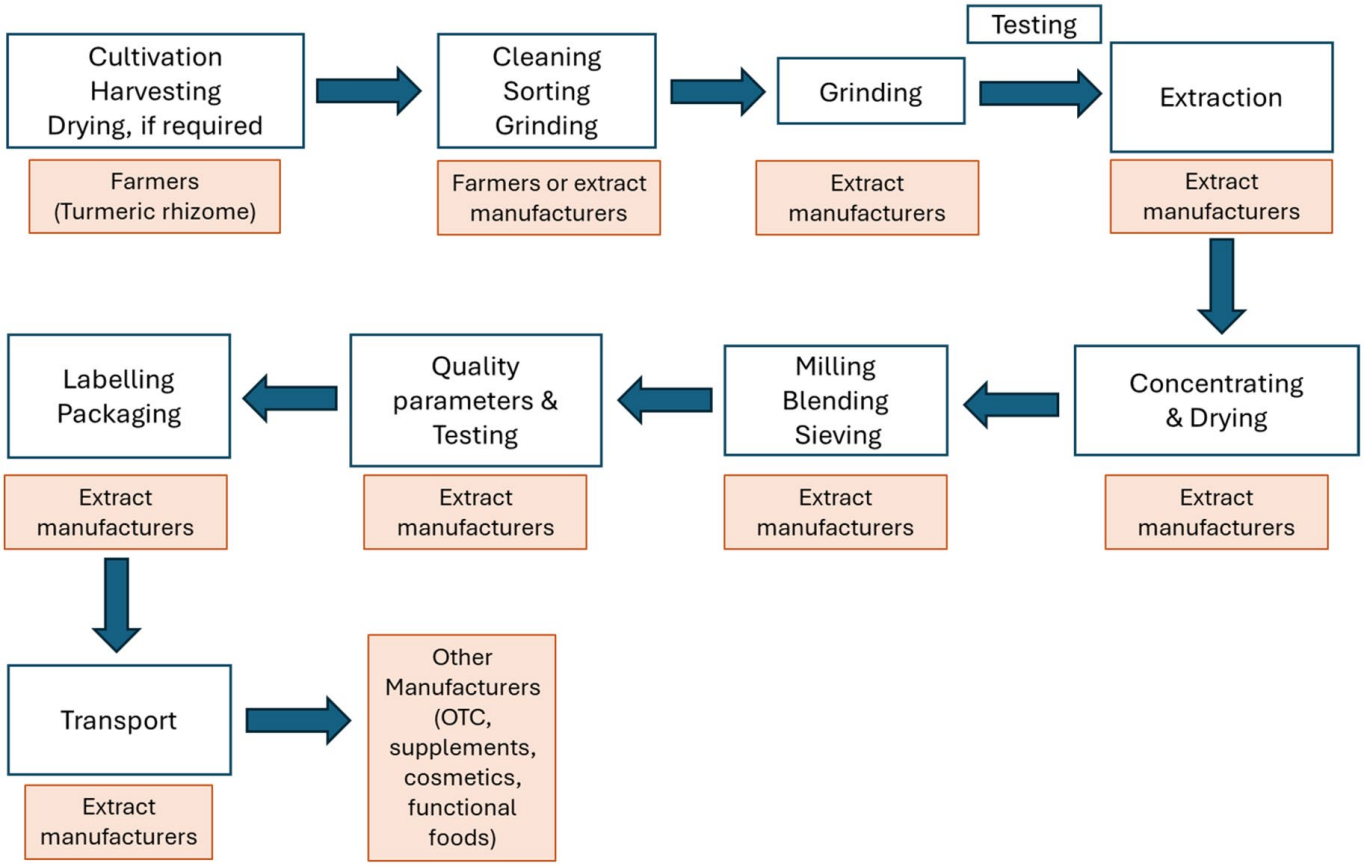
Turmeric supply chain for large botanical ingredient manufacturers (image provided by Aboli Girme, Natural Remedies, Bangalore, India).

Traders (buyers) can also sell the turmeric to extract manufacturers, which produce the ingredients that are commonly found in turmeric dietary or food supplements. Large turmeric manufacturers prefer to have a tighter control over the supply-chain to avoid price fluctuation and have better control of the quality. Such manufacturers usually give planting material to farmers; then it is harvested in the presence of a representative of the company, cleaned, boiled, and dried. The dry material is shipped to the manufacturing center, where grading is done based on information from root/rhizome analysis, and then the material goes to the processing site (Figure 2). These vertically integrated production systems, where the manufacturer purchases the turmeric directly from the farmer, mean that farmers may receive a higher price, and the company may garner higher profits due to the elimination of costs for the middlemen.

As with every agricultural commodity, quality is of utmost importance for consumer satisfaction. One of the pressing quality issues with turmeric quality is the problem of adulteration. Historically, turmeric powder was mostly known as an adulterant of other spices, especially mustard seed. A publication from 1904 on the composition and adulteration of ground mustard (*Brassica juncea* (L.) Czern., Brassicaceae) states that “coloring with turmeric was also a time-honored custom, and, in fact, still prevails”. (Leach 1904) In a subsequent paper, the same author notes that turmeric is “of chief interest to the analyst as an adulterant of other spices,” such as ginger (*Zingiber officinale* Roscoe, Zingiberaceae), cayenne pepper (*Capsicum annuum* L., Solanaceae), and mace (*Myristica fragrans* Houtt., Myristicaceae).

There is some evidence that turmeric adulteration with inorganic salts may date back to the nineteenth century, as intentional adulteration of curry with lead chromate was reportedly common practice at that time according to Power. (Power et al. 1969) The same author also mentions a 1948 report by the British Food Standards Committee that Indian traders treated turmeric with lead chromate to improve its color, leading to the “curry order” of 1949, in which the lead concentration in curry in the UK was limited to 10 ppm. (Power et al. 1969) Other synthetic dyes, such as metanil yellow, appear to have been used in the turmeric trade at least as far back as the 1960s, although publications documenting such use at the time are scarce. In addition to the admixture of undeclared dyes, Govindarajan and Stahl also mention adulteration of turmeric spice with undeclared bulking agents (starches from lower cost ingredients), exhausted turmeric root powder, and other *Curcuma* species. (Govindarajan and Stahl 1980) Substitution of turmeric with other *Curcuma* species was mentioned as early as 1950 in a book published in India according to Sen et al. (Sen et al. 1974). In their paper, the authors write that “adulteration of ordinary spices and condiments is exceedingly prevalent in India and probably the most subject to admixture is turmeric.” The addition of undeclared synthetic curcumin to turmeric extracts to create lower-cost ingredients that comply with the stated total curcuminoid concentrations in the dietary/food supplement market is a more recent development. To our knowledge, the first report of such extracts being marketed was published in 2011. (Watson 2011) This brief historical review provides evidence that turmeric adulteration is constantly evolving as fraudsters try to come up with new strategies to fool the most commonly used quality control assays. (Bejar 2018)

Starting in 2004, a growing number of publications and reports indicate that a substantial number of commercial turmeric products sold as spice, or dietary or food supplements, are adulterated. A recent review of turmeric adulteration, which included 15 publications and a total of 1247 commercial samples showed that the risk of adulteration is substantially higher (42.2%) for turmeric extracts and products containing extract and powder mixtures (34.8%) than for powdered turmeric (15.2%), suggesting that more highly processed ingredients and finished products have an increased risk of being adulterated. Overall, 206 of the 1247 samples (16.5%) were reported to be adulterated. (Orhan et al. 2024) The goal of this scoping review is to provide an insight into the differences of turmeric adulteration schemes, taking into consideration marketing claims, i.e., spice versus medicinal use, but also looking at differences in adulteration schemes based on geographical regions.

## Methods

**Figure 3.**
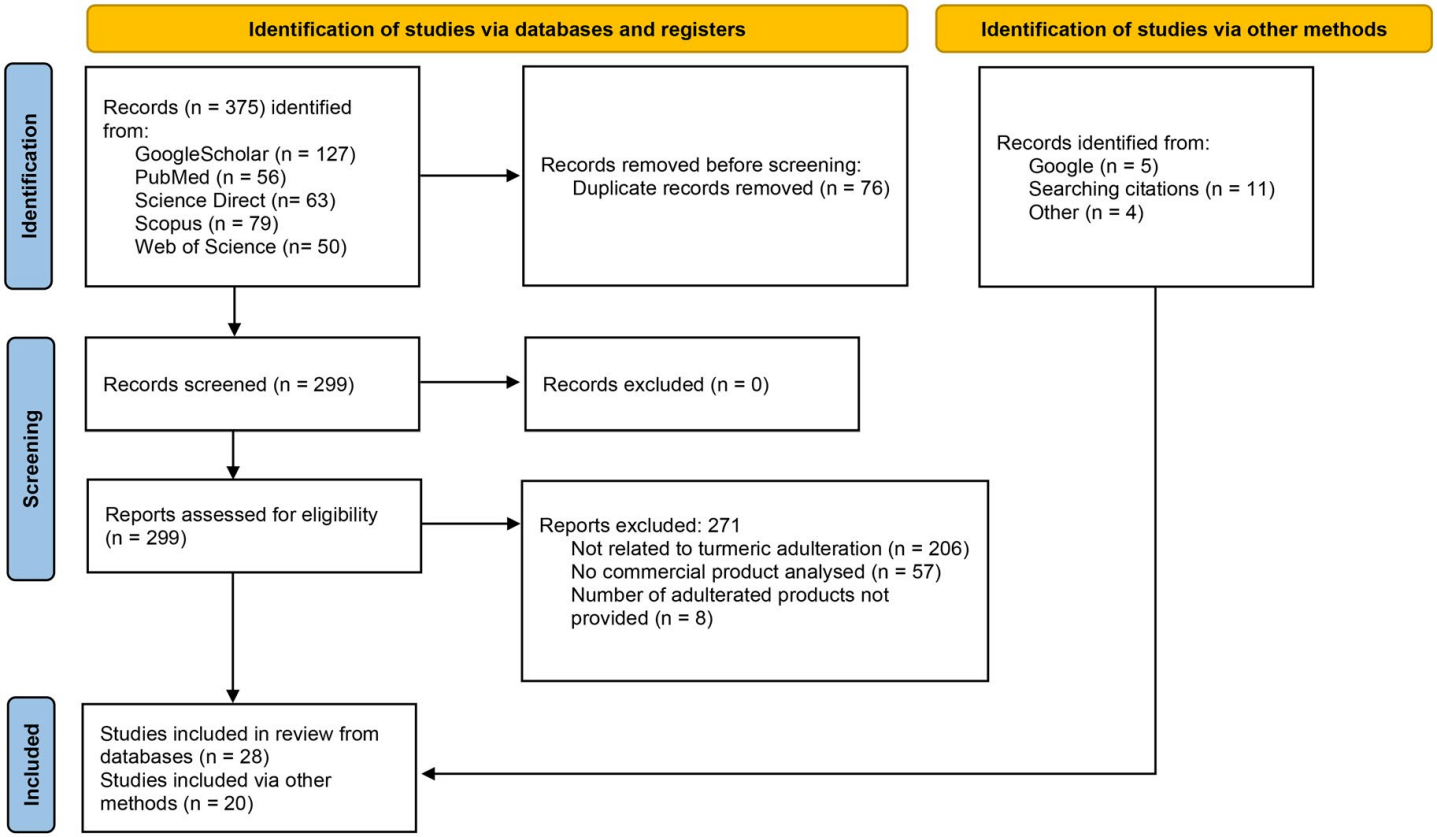
PRISMA flow diagram of the identification process for the 48 articles assessing the authenticity of commercial turmeric samples that were included in this scoping review.

This scoping review was based on Preferred Reporting Items for Systematic reviews and Meta-Analyses extension for Scoping Reviews: Checklist and Explanation (PRISMA-ScR) (Tricco et al. 2018). To identify potentially relevant documents, the systematic searches were conducted on five databases on November 7, 2025, using combinations of relevant keywords and Boolean operators: “turmeric” OR “Curcuma longa” AND “commercial products” AND “adulteration” AND NOT “saffron” for Google Scholar and Scopus, “turmeric” OR “Curcuma longa” AND “adulteration” for PubMed, “turmeric” AND “adulteration” AND “commercial products” AND NOT “saffron” for Science Direct, and “turmeric” AND “Curcuma longa” AND “commercial” for Web of Science. Three researchers (S.G., N.O., and Ç.K.) performed the literature search, and data extraction was performed by two researchers (S.G. and N.O.) independently. Any discrepancies were planned to be resolved by a third author if necessary. Inclusion criteria required that studies evaluate the authenticity of turmeric commercial products in any form, including whole or powdered rhizomes, extracts, dietary supplements, or herbal medicines. Scientific articles, government or company analysis reports, and theses were included if they were published between the period of 2000–2025, related to turmeric adulteration, and included commercial products. Studies were excluded if they did not align with the study’s conceptual framework or if they addressed only method development or single-adulterant analysis without evaluating commercial products. Bibliographies of all publications were additionally screened for relevant references to broaden the search. An additional 19 papers were found searching the citations or by using Google Search with the keywords “Curcuma longa”, “turmeric”, “government report”, and “adulteration”. One more publication from the CVUA (Chemisches und Veterinäruntersuchungsamt) Stuttgart was found using “Kurkuma”, “Verfälschung”, and “Report” as keywords (Figure 3).

All the data were tabulated using Google Sheets and analyzed with simple descriptive statistics (n; %). A data-charting form was jointly developed by two authors to determine the variables to be extracted. The two authors (S.G. and N.O.) charted the data together, discussed the results, and refined the data-charting form. Table 1 represents the details of the final dataset, including author names, year of publication, geographic location, number of adulterated and total commercial products, and adulteration rates by product form (spice or dietary supplement/herbal medicine). Results were grouped by geographical region and are discussed under the corresponding continent headings. In total, 48 papers were included in the review. For two publications, (Forsyth et al. 2024, Lerch and Bock 2024) only a subset of the total sample numbers was included in the review, since the authors determined adulteration on this subset only.

**Table 1.** Published reports and reported adulteration rates of commercial turmeric products.

Author(s)	Country/Continent	Number of Adulterated Samples/Number of Total Samples						
		Adulteration overall	Asia	India	Europe	North America	Spice	Dietary supplement and herbal medicine
(Sasikumar et al. 2004)	India	3/3	3/3	3/3	–	–	3/3	–
(Dixit et al. 2009)	India	105/712	105/712	105/712	–	–	105/712	–
(Dhanya et al. 2011)	India	4/6	4/6	4/6	–	–	4/6	–
(Avula et al. 2012)	USA	0/6	–	–	–	0/6	–	0/6
(New Zealand Ministry for Primary Industries 2012)	New Zealand	0/16	–	–	–	–	0/16	–
(Booker et al. 2014)	China (4), India (7), Taiwan (1), UK (5), USA (5)	12/22	4/12	2/7	5/5	3/5	–	9/10
(Amel 2015)	Algeria	3/15	–	–	–	–	3/15	–
(Nath et al. 2015)	India	29/88	29/88	29/88	–	–	29/88	–
(Parvathy et al. 2015)	India	1/10	1/10	1/10	–	–	1/10	–
(Mudge et al. 2016)	Canada	4/12	–	–	–	4/12	–	4/12
(Osathanunkul et al. 2017)	Thailand	6/7	6/7	–	–	–	6/7	–
(Skiba et al. 2018)	USA	5/35	–	–	–	5/35	–	5/35
(Mosa et al. 2018)	UAE	2/3	2/3	–	–	–	2/3	–
(Chatzinasiou et al. 2019)	Germany, UK, USA	11/56	–	–	–	–	–	11/56
(Canadian Food Inspection Agency 2019)	Canada	0/15	–	–	–	0/15	0/15	–
(Girme et al. 2020)	India	4/16	4/16	4/16	–	–	–	4/16
(Zhang et al. 2019)	China	4/4	4/4	–	–	–	4/4	–
(Menniti-Ippolito et al. 2020)	Italy	11/18	–	–	11/18	–	0/1	11/17
(Rodrigues et al. 2020)	Brazil	9/29	–	–	–	–	9/29	–
(Oh and Jang 2020)	South Korea	0/10	0/10	–	–	–	0/10	–
(Sahu et al. 2020)	India	3/27	3/27	3/27	–	–	3/27	–
(Kim et al. 2021)	Italy	2/2	–	–	2/2	–	–	2/2
(Kuruldak et al. 2021)	Türkiye	0/15	–	–	0/15	–	0/15	–
(Maquet et al. 2021)	Europe	28/316	–	–	28/316	–	28/316	–
(DE Sales Mélo et al. 2021)	Brazil	5/10	–	–	–	–	5/10	–
(Rao et al. 2021)	India	2/9	2/9	2/9	–	–	2/9	–
(Sheu et al. 2021)	Taiwan	0/5	0/5	–	–	–	0/5	–
(Vostrikova et al. 2021)	Russia	10/10	–	–	10/10	–	10/10	–
(Canadian Food Inspection Agency 2021)	Canada	0/22	–	–	–	0/22	0/22	–
(NOW FOODS 2021)	USA	5/25	–	–	–	5/25	–	5/25
(You et al. 2022)	USA	5/14	–	–	–	5/14	–	5/14
(Sorng et al. 2022)	France	2/30	–	–	2/30	–	–	2/30
(Brusač et al. 2022)	Asia (5), Europe (23), North America (7), unknown (1)	9/36	4/5	–	3/23	2/7	–	8/34
(Verma et al. 2022)	India	2/20	2/20	2/20	–	–	2/20	–
(Siudem et al. 2023)	Poland	1/4	–	–	1/4	–	–	1/4
(Mishra et al. 2023)	India	0/7	0/7	0/7	–	–	–	0/7
(Sadef et al. 2023)	Pakistan	63/233	63/233	–	–	–	63/233	–
(Harke et al. 2023)	India	0/6	0/6	0/6	–	–	0/6	–
(Liu et al. 2023)	USA	1/12	–	–	–	1/12	–	1/10
(Ullah et al. 2023)	Pakistan	4/5	4/5	–	–	–	4/5	–
(Amsaraj et al. 2024)	India	4/16	4/16	4/16	–	–	4/16	–
(Forsyth et al. 2024)	India	6/75	6/75	6/75	–	–	6/75	–
(Lerch and Bock 2024)	Germany	2/32	–	–	2/32	–	–	2/32
(Yang et al. 2024)	South Korea	0/22	0/22	–	–	–	–	–
(Wei et al. 2025)	China	10/98	10/98	–	–	–	–	–
(Singh et al. 2024)	USA	0/15	–	–	–	0/15	–	0/15
(Moravcová et al. 2025)	Czech Republic	3/7	–	–	3/7	–	–	3/7
(Payn et al. 2025)	Sri Lanka	68/79	68/79	–	–	–	68/79	–
**Total**		**448/2235**	**328/1478**	**165/1002**	**67/462**	**25/168**	**361/1767**	**73/332**
**Adulteration Percentage**		**20.04%**	**22.19%**	**16.47%**	**14.50%**	**14.88%**	**20.43%**	**21.99%**

The extent of adulteration was determined based on the assessment of the authors of the published papers, and – in some cases – by the authors of this study. When the study authors assessed turmeric extract adulteration, the presence of synthetic curcumin was concluded when the concentration of curcumin was above 90% of the sum of all curcuminoids (curcumin, demethoxycurcumin, and bisdemethoxycurcumin) based on the results of You et al. which proposed an upper limit of 85.4% curcumin for biobased turmeric extracts. (You et al. 2022) Our approach is in line with the 90% relative curcumin content threshold used by Skiba et al. to determine products containing synthetic curcumin. (Skiba et al. 2018) Other quality-related parameters, for example the evaluation of compliance of curcuminoid contents in powdered turmeric with national or international standards, or an evaluation if dietary supplement products met curcuminoid standardization claims, were beyond the scope of this review. Since the goal of this scoping review was to include as much data on turmeric adulteration as possible, the lack of using a scientifically valid method was not used as a criterion for excluding publications.

## Results

**Figure 4.**
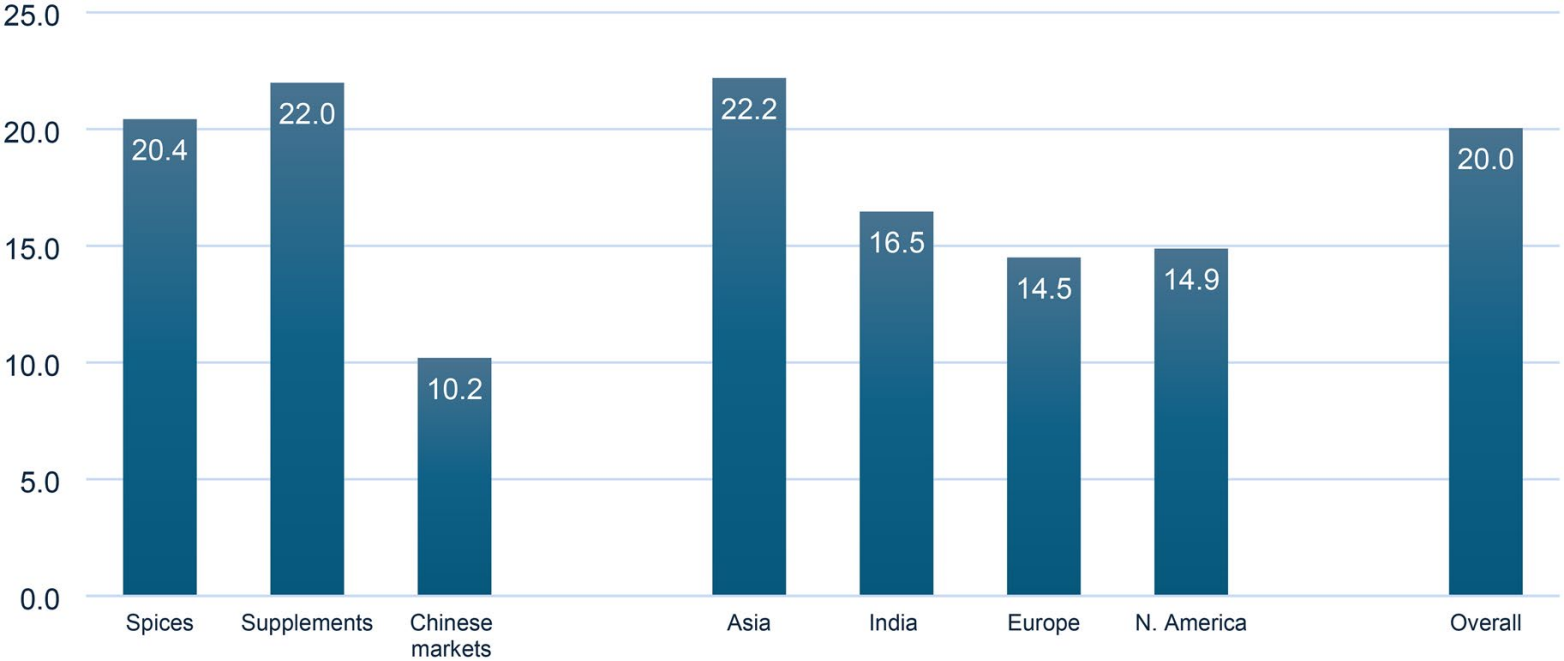
Adulteration percentage of turmeric products in different markets across the globe. Supplements: dietary and food supplements.

A total of 48 publications representing 2235 commercial turmeric samples were included in the study. Of these samples, 1767 were categorized as spices, 332 as food supplements, dietary supplements, or herbal medicines, and 136 samples were not assigned to any category. Ninety-eight (98) of the unassigned samples were from herbal markets in China, 22 from herbal markets in South Korea, and the remaining 16 samples could not be assigned to any category due to missing information. The overall adulteration rate was 20.0%, with spice samples having a slightly lower percentage of adulterated samples (20.4%) than dietary and food supplements (22.0%). Of the 98 samples sourced from herbal markets in China, 10 (10.2%) were reportedly adulterated. (Wei et al. 2025) (Figure 4; Table 1).

The majority of samples were purchased in Asia (*n* = 1478), followed by Europe (*n* = 462), North America (*n* = 168), South America (*n* = 39), Oceania (*n* = 16), and Africa (*n* = 15). Close to half of all samples (*n* = 1002) were obtained in India. Samples from one publication (*n* = 56) purchased from the United Kingdom, Germany, and the USA were not assigned to any geographical region since the authors did not specify how many samples originated from a specific country, (Chatzinasiou et al. 2019) and in one publication, one sample was of unknown origin. (Brusač et al. 2022) Of the geographic regions where results of more than 100 samples were available, Asia (22.2%) had higher adulteration rates than Europe (14.5%) and North America (14.9%). The extent of adulterated turmeric samples was lower in India (16.5%) than the 22.2% calculated for all of Asia (Figure 4). Adulteration rates in South America, Africa, and Oceania were 35.9%, 20.0% and 0%, respectively, but the very low number of samples does not allow any general conclusion of the authenticity of turmeric products sold on these three continents.

**Figure 5.**
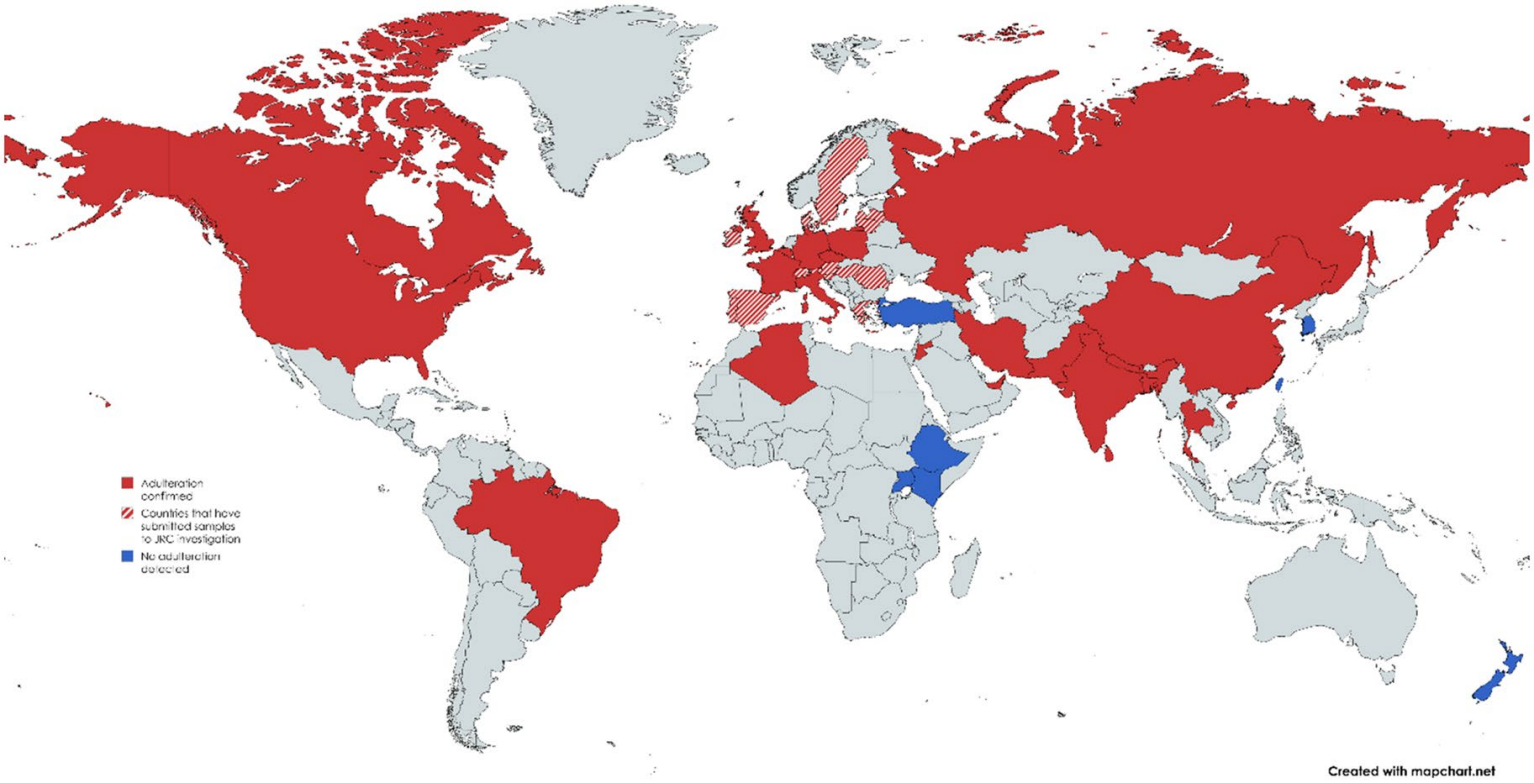
Countries with evidence of turmeric adulteration (red) in the scientific literature, for which adulteration is possible (red and white) based on results from the European Commission’s Joint Research Centre (JRC) Technical Report, (Maquet et al. 2021) and for which all tested samples were considered authentic (blue).

Overall, adulteration of turmeric products has been documented in 22 countries so far (excluding countries in Europe which submitted samples for analysis to the European Commission’s Joint Research Center [JRC] (Maquet et al. 2021) as part of an EU initiative to establish the prevalence of adulterated spice samples on the European market, since the results were not presented on a country-by-country basis) (Figure 5).

**Figure 6.**
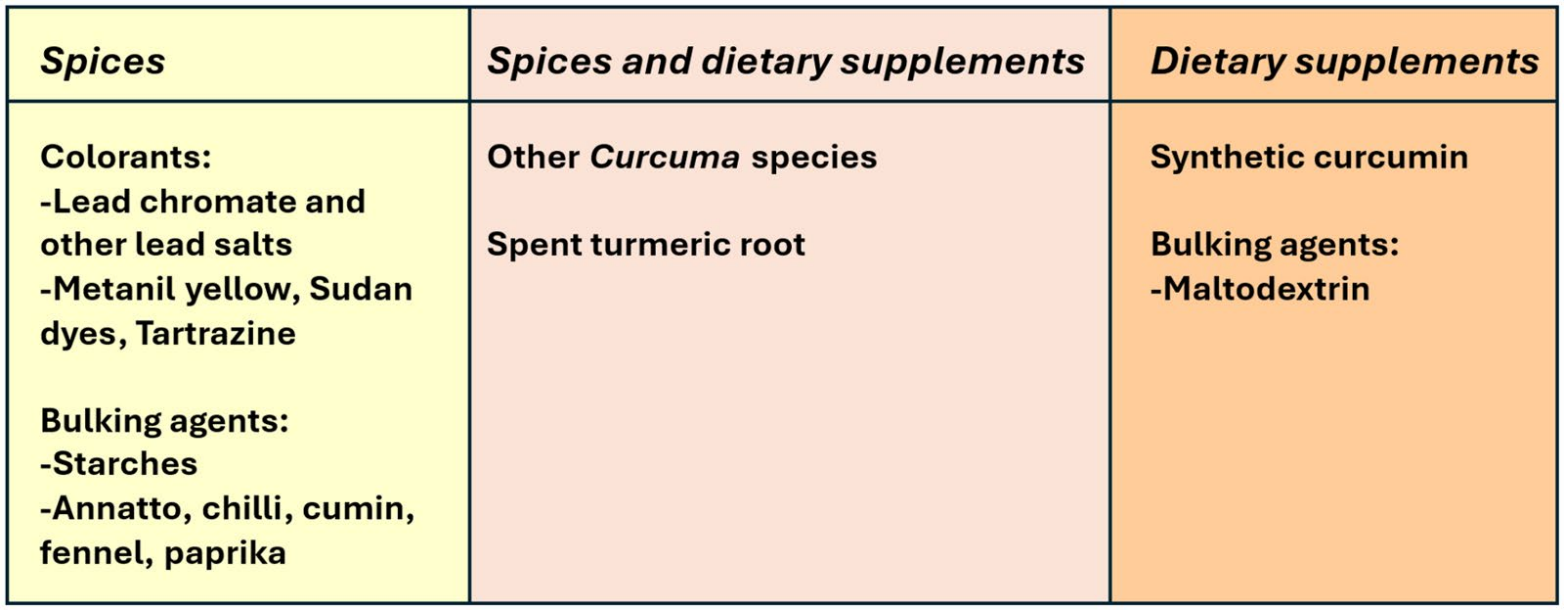
Different adulteration methods for turmeric depending on the marketing category/channel of trade.

Turmeric is adulterated in numerous different ways. For turmeric spice, the undeclared presence of synthetic dyes, including lead chromate, metanil yellow, Sudan dyes, and tartrazine, is the main concern due to the toxicity of some of these dyes. Other adulterants of turmeric spice are natural colorants such as chili or paprika powders (*Capsicum* spp., Solanaceae), diluents like barley (*Hordeum vulgare* L., Poaceae), cassava (*Manihot esculenta* Crantz, Euphorbiaceae), corn (*Zea mays* L., Poaceae), oat (*Avena sativa* L., Poaceae), rice (*Oryza sativa* L., Poaceae), rye (*Secale cereale* L., Poaceae), or wheat (*Triticum* spp., Poaceae) starch, powdered fennel (*Foeniculum vulgare*, Apiaceae) or cumin (*Cuminum cyminum* L., Apiaceae), or substitution with other *Curcuma* species (*C. zedoaria* (Christm.) Roscoe*, C. elata* Roxb.). In the case of food or dietary supplements, adulteration occurs mainly with synthetic curcumin or by excessive dilution with inert materials. (Figure 6).

Adulteration with chalk or yellow soapstone, which has been alleged in the literature from India, was not found in any of the published papers. The detection of undeclared natural or synthetic colorants was the objective in 18 studies, while the authors of 12 determined the presence of diluents. Also, 11 investigations looked into the presence of other *Curcuma* species or non-specified plant adulterants, while 18 publications (including ten publications assessed by the authors of this study) verified the presence of synthetic curcumin, respectively. However, the number of publications on a specific adulterant is unlikely to be representative of the extent of an adulteration problem, since investigations may be prompted by other factors, e.g., a particular health concern such as the potential toxicity due to lead chromate in powdered turmeric. Therefore, the available data do not permit any conclusions about the frequency of use of specific adulterants. A list of the 48 papers included in this study is provided in Table 1.

## Geographical differences in adulteration

Based on the available information, there appear to be some distinct differences in the way that turmeric is adulterated depending on the geographic region and the regulatory environment in which turmeric is sold. As an example for the latter, standards for powdered turmeric vary from region to region. The United States Pharmacopeia requires a minimum content of 3% total curcuminoids and 3% essential oil, while the European Pharmacopeia specifies a minimum of 2%, and a minimum of 2.5% essential oil. (United States Pharmacopeia 2025, European Pharmacopoeia 11.0, 2024) On the other hand, the Chinese Pharmacopeia requires a minimum of 7% volatile oil, and 1% curcumin without specification for total curcuminoids. (Pharmacopoeia of the People’s Republic of China, 2020) For spices, the minimum total curcuminoid content is usually 2%. (International Organization for Standardization (ISO) 1983; Bureau of Indian Standards, 2010) Interestingly, in their “Spices Grading and Marking Rules “the Government of India allows for two grades of turmeric to be sold as spice: The standard grade must contain a minimum of 2% curcuminoids, while the special grade contains at least 3% total curcuminoids. (Government of India 2012) However, in some areas, specifications for curcuminoid contents in turmeric spice are lacking. (Singh et al. 2021, European Spice Association 2003, American Spice Trade Association 2008) Hence the specific standard applicable in a country or region may impact the quality of turmeric products available on the market.

While there are fairly large datasets on the authenticity of turmeric available from certain areas, information from Africa, South America, and Oceania is scarce, making an overall assessment of the turmeric quality in these markets challenging.

### Asia

#### Southern Asia

With 14 publications covering 1002 samples, India is the country with the single largest set of data on the authenticity of turmeric. The vast majority of the Indian products analyzed (*n* = 972) were spice samples. Most often, adulteration with dyes, mainly metanil yellow, was investigated. Of the 947 commercial Indian samples tested for metanil yellow, 150 (15.8%) were positive. Amsaraj et al. and Forsyth et al. reported adulteration with lead chromate in India. Based on Forsyth et al. such adulteration is particularly frequent in the Indian states of Bihar and Assam, but also in the neighboring countries of Bangladesh, Pakistan, and Nepal. (Forsyth et al. 2019a, Forsyth et al. 2024, Forsyth et al. 2019b, Amsaraj et al. 2024) Since the number of samples adulterated with lead chromate was not provided by Forsyth et al. these samples are not included in Table 1. After the initial publication connecting elevated blood lead levels in Bangladesh with intake of lead chromate adulterated turmeric, (Forsyth et al. 2019a) the Bangladesh Food Safety Authority implemented a series of actions, including a public notice declaring that turmeric adulteration was a prosecutable act, warning consumers, businessmen, and people working in the polishing mills of the dangers of lead, imposing fines on traders found to be treating turmeric roots with the dye, and training of employees of the Bangladesh Food Safety Authority on a handheld x-ray fluorescence analyzer (XRF) to detect lead and chromium in turmeric samples sold across Bangladesh. Remarkably, the number of lead-contaminated turmeric samples decreased from 47% in 2019, to 5% in the beginning of 2020, 2.3% in the fall of 2020, and 0% in the beginning of 2021. (Forsyth et al. 2023)

Adulteration with another type of colorant, Sudan dye, was reported from Pakistan in 28.2% of the 238 samples that were evaluated. (Sadef et al. 2023, Ullah et al. 2023) The presence of unidentified dyes was reported in 68 of 79 turmeric spice samples from Sri Lanka based on results obtained by wet chemical methods. (Payn et al. 2025) Other types of adulteration reported in turmeric spice from Southern Asia include substitution with *C. zedoaria *and the presence of undeclared bulking agents such as cassava starch, rye, and wheat. (Dhanya et al. 2011, Sasikumar et al. 2004, Parvathy et al. 2015) Osathanunkul et al. submitted seven commercial turmeric spice samples from stores in Thailand to Barcode High Resolution Melting analysis and found that six of the samples contained undeclared other plants. However, the identity of the adulterants was not disclosed. (Osathanunkul et al. 2018)

The number of publications detailing results from investigations into the authenticity of dietary supplement or herbal medicine samples from Southern Asia is comparatively low. Girme et al. found a byproduct of the curcumin synthesis in four of 16 samples from India. (Girme et al. 2020) Adulteration with synthetic curcumin in a sample from Tamil Nadu was also evident based on an assessment of the HPTLC chromatograms in a 2014 publication by Booker et al. (Booker et al. 2014) On the other hand, no adulteration with synthetic curcumin was seen by Mishra et al. and all seven samples in the investigation were free from Sudan dyes. (Mishra et al. 2023) The latter paper reported that one product contained higher concentrations of bisdemethoxycurcumin than curcumin and hence was “adulterated”. While higher concentrations of bisdemethoxycurcumin compared to curcumin are unusual, other publications (Brusač et al. 2022, Sorng et al. 2022) have also reported that curcumin may not be the dominant curcuminoid in commercial products. In addition, some of the potential adulterant species, e.g., *C. aromatica* Salisb. (syn. *C. wenyujin* Y.H. Chen & C. Ling), *C. zanthorrhiza* Roxb., and *C. zedoaria*, also contain curcumin as the most prominent curcuminoid, (Cardellina 2020) while potentially confounding *Curcuma* species from China contain little to no curcuminoids. (Wei et al. 2025) Therefore, samples containing a slightly higher bisdemethoxycurcumin or demethoxycurcumin concentration compared to curcumin are not necessarily adulterated.

#### China

There is much less information about the authenticity of turmeric products sold in China compared to India. Some data could have been missed since the search excluded papers published in Chinese. Only one publication was retrieved that reported results on commercial turmeric spice sold in China, despite its frequent use in Chinese cuisine. In this investigation, two of the four spice samples were adulterated with corn, and the other two were diluted with fennel. (Zhang et al. 2019) The largest investigation determined the authenticity of crude turmeric roots or rhizomes obtained from herbal markets. According to the authors, the Chinese Pharmacopeia permits the use of roots of *C. longa*, *C. aromatica*, *C. kwangsiensis* S.G.Lee & C.F.Liang, and *C. phaeocaulis* as Curcumae rhizoma. Using a number of orthogonal methods, the authors determined that ten of the 98 samples were adulterated with *C. elata*. One of the adulterated samples was labeled as C. *phaeocaulis* rhizome, while the other nine were labeled as Curcumae rhizoma. (Wei et al. 2025) A different type of adulteration was evidenced by Booker et al. (Booker et al. 2014) Two of the samples that originated in China were deemed to be adulterated: One did not contain any of the typical turmeric constituents, while the other one was primarily composed of curcumin and hence is believed to be of synthetic origin. Both of these samples were sourced on TCM markets in the UK, but since they were knowingly manufactured in China, these samples are listed as Chinese samples in this review.

#### Japan and South Korea

While there was no information obtained on the authenticity of turmeric in Japan, two publications were retrieved that detailed the results of commercial turmeric samples sold in South Korea. The first investigation evaluated the presence of corn, rice, and wheat starch in ten commercial spice samples by real time PCR. Starch was absent in all of the products. (Oh and Jang 2020) Yang et al. (Yang et al. 2024) did a screen for nine azo dyes in 22 products obtained from Gyeongdong Oriental Market (Seoul, Korea), Daegu Oriental Market (Gyeongsang Province, Korea), and Jecheon Oriental Market (Chungcheong Province, Korea). No azo dye was found in any of the samples. Since these markets sell both spices and traditional medicinal ingredients, it is not clear if these products were sold for culinary or medicinal purposes. While the data suggest a low adulteration rate in South Korea, the small sample numbers and limited number of analytes make it difficult to assess the authenticity of turmeric samples in these markets. However, the relatively stringent regulations regarding food and medicine in Japan and South Korea may lead to a lower risk of adulterated turmeric products finding their way into the markets.

#### Middle East

Adulteration of turmeric spice has been reported in three publications. Mosa et al. used DNA barcoding to assess four turmeric samples, including three powders and a fresh rhizome, from local herbal markets in the United Arab Emirates. Two samples yielded DNA barcodes for *C. phaeocaulis*, while one sample did not provide useful DNA sequences. The fourth sample was considered authentic. A paper assessing the quality of spices sold at different herbal shops in Jordan reported that the undeclared addition of coloring agents was observed in some of the five turmeric samples. However, the exact number of adulterated samples was not provided. (Abu-Hamdah et al. 2005) Tamiji et al. used FT-IR and multivariate statistics to detect adulteration with wheat flour, pistachio (*Pistacia vera*, Anacardiaceae) hull waste, and dry bread powder in 102 turmeric samples purchased from supermarkets in Tehran, Iran. While the exact number of adulterated samples is not provided, the authors indicate that a portion of the analyzed products was adulterated with dry bread powder. (Tamiji et al. 2022) Despite the limited amount of available information, it appears that the risk of purchasing adulterated turmeric in some Middle Eastern countries is relatively high.

## Africa

Africa is one of the continents for which the least amount of information about the authenticity of turmeric products has been published. A publication from Algeria indicates adulteration of at least three of the fifteen samples analyzed by microscopy. Adulteration was based on the presence of calcium oxalate crystals in some samples, but the authors were unable to identify the adulterating materials. A review on the importance of Africa as a turmeric supplier noted that information on adulteration with food colorants is generally lacking, but that some respondents in a survey from Ghana allegedly heard that turmeric is adulterated with metanil yellow. (Abia et al. 2025) A study assessing lead levels in turmeric and curry samples from Ethiopia, Kenya, and Uganda concluded that adulteration with lead chromate was nonexistent. (Woldetsadik et al. 2023) Unfortunately, the authors did not specify the number of turmeric samples in the study; hence it was omitted from Table 1. While there is no robust evidence for the undeclared addition of synthetic dyes to turmeric in Africa, there is also a lack of information on the quality and authenticity of the marketed products. One of the issues raised by Abia et al. (Abia et al. 2025) is the lack of harmonized regulations regarding turmeric across Africa, and the lack of limits for heavy metals such as lead in some African countries. Therefore, it appears that there is a relatively low risk of regulatory enforcement for those who sell adulterated turmeric on African markets.

## Australia and New Zealand

Although turmeric didn’t make it into the top 10 most popular herbs and spices in Australia in 2024, (Mantzioris et al. 2024) data on the use of turmeric as a spice in Australia indicate that it is quite popular. A survey of 1023 adult Australians published in 2014 indicated that curry (of which turmeric is one of the main ingredients) was used by 78.9% of the respondents, while turmeric was used by 55.5%. (Wang and Worsley 2014) Despite the relatively high use of turmeric in Australia, data on its authenticity could not be found. On the other hand, the New Zealand Ministry for Primary Industries performed an analysis of imported spices, including 16 samples of turmeric. One of the tests was evaluating the presence of unauthorized dyes in the turmeric samples, but no such dyes were present. (New Zealand Ministry for Primary Industries 2012)

Hoban et al. used a combination of HPLC-quadrupole time-of-flight mass spectrometry (HPLC-qToF-MS), HPLC-UV/Vis, GC-MS, and DNA barcoding with next-generation sequencing to verify the presence of adulterants and contaminants in 49 herbal medicines sold in Australia used to treat inflammatory conditions. The authors did not provide a list of the samples that were analyzed, but one turmeric product was deemed to be adulterated with wooly prince’s plume (*Stanleya tomentosa* Parry, Brassicaceae). (Hoban et al. 2020) Since the native range of wolly prince’s plume is limited to the US states of Wyoming, Montana, and Idaho, the plant is relatively uncommon, and the amount of the plant in the sample was not determined, this is most likely a case of accidental contamination with wolly prince’s plume DNA rather than intentional adulteration. Overall, the available data do not permit to assess the prevalence of adulteration of turmeric products on the markets in Australia and New Zealand.

## Europe

A large investigation into the authenticity of turmeric spice sold in Europe was carried out by the European Commission’s Joint Research Center (JRC). (Maquet et al. 2021) The investigation tested 316 turmeric spice samples from 22 European countries for the presence of non-authorized dyes, fillers, and compliance with ISO specifications regarding the curcuminoid content. Twenty-four samples were considered to be adulterated due to the content of paprika/chili (*Capsicum* spp., Solanaceae) and starch-containing species such as corn, rice, and other cereals (*Avena* spp./*Triticum* spp., Poaceae) above 2%. Three samples contained non-authorized dyes, and one sample was an extract rather than a powder. The JRC report lists an additional six samples as adulterated since these samples did not contain the minimum 2% curcuminoid content required by the ISO 5562:1983 standard. (International Organization for Standardization (ISO)), 1983) However, these six samples were not considered to be adulterated by the authors of this scoping review since they do not represent the unintentional or fraudulent addition of non-authentic substances or removal or replacement of authentic substances without the purchaser’s knowledge. (Food Chemical Codex 2016). The only other paper on the quality of European turmeric spices was published by Vostrikova et al. These authors found that all of the ten samples obtained from markets in Moscow and Smolensk, Russia, were adulterated with lead chromate or other chromates, or diluted with starches. (Vostrikova et al. 2021)

Several authors investigated the quality of turmeric food supplements sold in Central and Western Europe. Investigations from France, Germany, Italy, and Poland all reported the sale of synthetic curcumin labeled as turmeric, with 17 of 82 tested samples being considered to contain synthetic turmeric. (Menniti-Ippolito et al. 2020, Sorng et al. 2022, Kim et al. 2021, Lerch and Bock 2024, Siudem et al. 2023) The presence of synthetic curcumin was also evidenced in products from Belgium (Brusač et al. 2022) and the Czech Republic, (Moravcová et al. 2025) and appears to be the most prominent issue currently with food supplements in Europe. The sale of synthetic curcumin labeled as turmeric extracts may be particularly prominent in Italy, since 13 of the 20 samples tested contained synthetic curcumin. (Kim et al. 2021, Menniti-Ippolito et al. 2020) However, the same product, of which two batches were tested by Kim et al. may also have been evaluated by Menniti-Ippolito et al. These Italian samples were analyzed as part of a broader investigation into turmeric food supplement composition after more than 20 case reports linking turmeric food supplement intake to liver injury. Other potential issues with turmeric authenticity were reported by Booker et al. who detailed two commercial samples that were made with an incorrect *Curcuma* species and three instances of exhausted turmeric being sold on markets in the UK. (Booker et al. 2014)

## North America

Contrary to other geographic regions, the authenticity of turmeric as a spice has not been widely investigated in North America. Two publications by the Canadian Food Inspection Agency, testing a total of 37 turmeric spice samples for the presence of lead chromate, were the only information retrieved for this scoping review. A 2017 paper revealed lead concentrations of 32 turmeric spice samples obtained from mainstream grocery stores, specialty stores, and ethnic markets throughout the greater Boston area. Half of the samples contained lead concentrations above 0.1 ppm (1 μg/g), which was chosen as the limit based on the maximum permissible lead concentration in candy. Two samples had very high lead concentrations (34.78 and 99.50 ppm), apparently due to the presence of lead oxide. (Cowell et al. 2017) It is not clear if the lead oxide stems from environmental contamination or from intentional adulteration.

Similar to the situation in Europe, the sale of synthetic curcumin labeled as turmeric extract appears to be the dominant issue for turmeric dietary supplements. At least five publications provide evidence to that effect with adulteration rates between 8.3 and 35.7%. (NOW FOODS 2021, You et al. 2022, Skiba et al. 2018, Mudge et al. 2016, Liu et al. 2023) Additionally, cases of excessively diluted products were reported. (NOW FOODS 2021) However, the most recent publication assessing the quality of turmeric dietary supplements, which used UHPLC-UV/Vis to determine curcuminoid concentrations, GC-MS for the quantification of the volatile constituents *ar*-turmerone, α-turmerone, and β-turmerone (curlone), and UHPLC-MS to detect any unexpected adulterants, did not find any adulteration in the 15 test samples. (Singh et al. 2024)

## South America

Brazil is the only South American country where published information on the quality of turmeric products could be found. The two publications retrieved described assays to determine the presence of undeclared starches (corn) in turmeric spice. (Rodrigues et al. 2020, DE Sales Mélo et al. 2021) Additionally, some samples contained undeclared annatto (*Bixa orellana* L., Bixaceae) or cumin. Based on these two publications, adulteration of turmeric spice may be relatively common in Brazil.

## Analytical test methods used to detect adulteration

**Figure 7.**
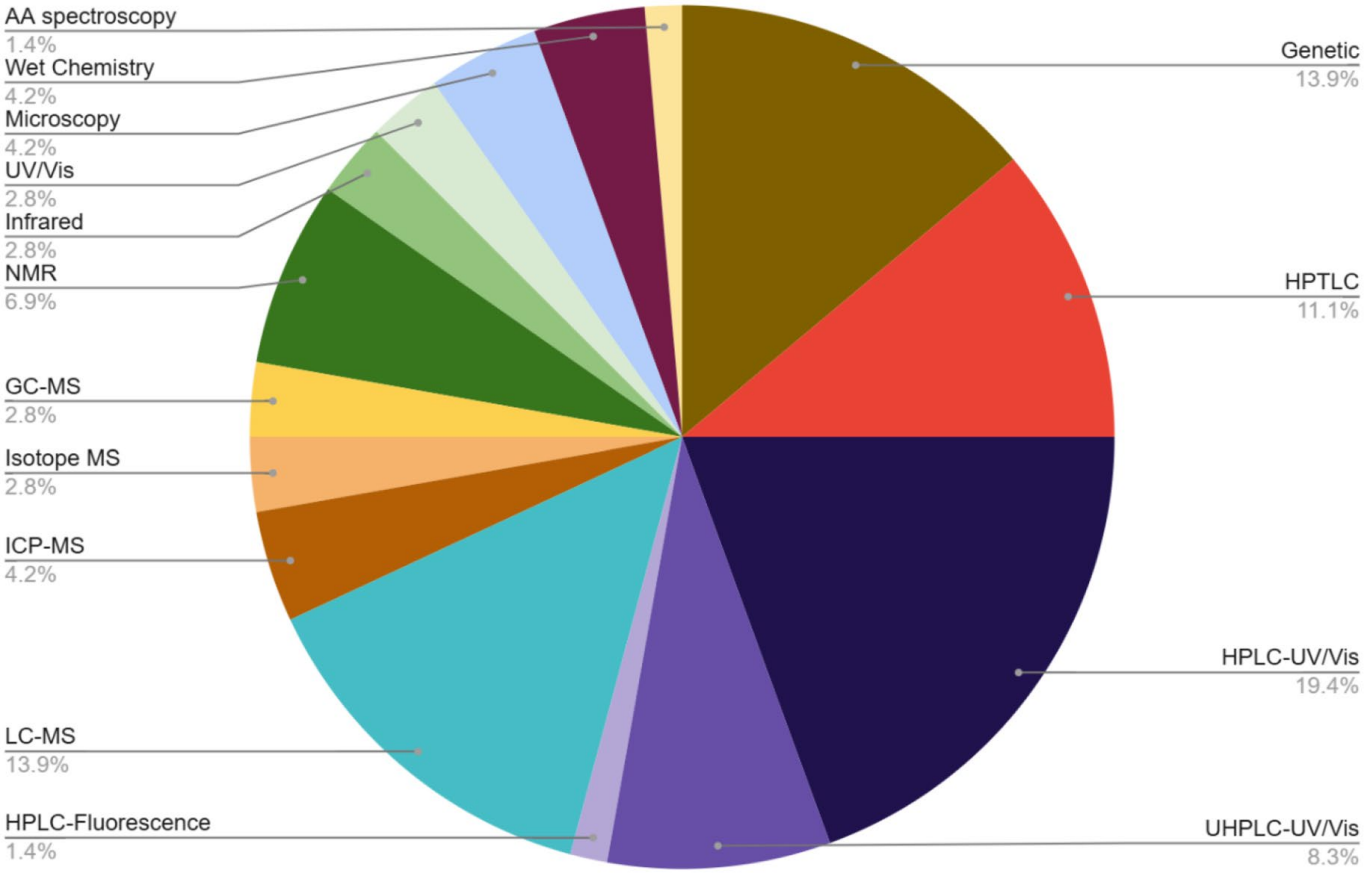
Analytical techniques used to determine turmeric authenticity. AA: atomic absorption; UV/Vis: ultraviolet/visible; NMR: nuclear magnetic resonance; GC-MS: gas chromatography – mass spectrometry; ICP-MS: inductively coupled plasma – mass spectrometry; LC-MS: liquid chromatography – mass spectrometry; HPTLC: high-performance thin-layer chromatography; HPLC: high-performance liquid chromatography; UHPLC: ultrahigh-performance liquid chromatography.

A majority of the publications reviewed focused on a particular type of adulteration, e.g., the presence of artificial colorants or excessive amounts of starch in turmeric spice, or the substitution of turmeric extracts with synthetic curcumin in dietary and food supplements. Most often (41.6%), HPLC or UHPLC was chosen for the analysis with UV/Vis or MS detectors. The authors used a genetic method or TLC/HPTLC, respectively, in 13.9% and 11.1% of the cases. Nuclear magnetic resonance was used in 6.9% of all the different analytical approaches, while other instrumental approaches, wet chemistry, and microscopy were less commonly used (Figure 7). In a bit more than half of the papers (*n* = 27), a single method was used, while in 19 papers, the authors preferred a combination of two or more tests. The two reports by the Canadian Food Inspection Agency did not include information about the test method. (Canadian Food Inspection Agency 2021, Canadian Food Inspection Agency 2019) A majority (68%) of authors assessing turmeric authenticity with an HPLC or UHPLC method used a validated method (*n* = 12), a method published by an official standard setting organization (*n* = 2), or used a contract laboratory (*n* = 3). In the remaining HPLC/UHPLC publications (*n* = 8), no validation data were reported. Compendial HPTLC or TLC methods were used in 3 publications, while two HPTLC methods were used as orthogonal assay in combination with a validated HPLC/UHPLC method. The methods in the remaining three papers including HPTLC or TLC data were either sent to a contract laboratory (*n* = 1) or were not validated (*n* = 2). Validation of genetic test methods was done in two cases, while for the remaining DNA-based methods, the extent of validation was unclear (*n* = 4) or the method was not validated (*n* = 4).

Each of the analytical test methods has its strengths and limitations. The advantages and disadvantages of analytical test methods for botanical ingredients have been the topic of numerous published articles, and those interested in the topic are referred to the relevant literature. (Smillie and Khan 2010, Lindenmaier et al. 2025, Upton et al. 2020, Parveen et al. 2016, Ivanova et al. 2016, Cardellina 2020).

## Discussion

The authenticity of turmeric products, both as a spice and dietary/food supplement, has been subject of many publications. An earlier review by the present authors, including 1247 commercial turmeric samples, found an adulteration rate of 16.5%, (Orhan et al. 2024) which is lower than the 20.0% calculated in this review. This paper includes 33 additional publications and 988 more samples. It provides a more comprehensive review of the turmeric trade and adulteration issues globally. The prior assessment by Orhan et al. suggests that extracts have a higher adulteration than extract/powder mixtures, with powders being the least likely to be adulterated. The percentages of adulterated samples with regard to Europe is also similar in the two reviews. However, Orhan et al. found a higher adulteration rate in North America (21.5%) compared to the 14.9% here. This discrepancy is mainly due to the inclusion of two reports from the Canadian Food Inspection Agency and a 2025 paper by Singh et al. on turmeric dietary supplements sold in the USA, which did not find any adulteration in the tested products. On the other hand, the estimated adulteration rate in Asia was lower in the 2024 review (16.8%) (Orhan et al. 2024) than the 22.2% reported in this study. This change can be explained by a large part due to the inclusion of two papers from Pakistan and Sri Lanka in this review with large sample numbers that reported a relatively high percentage of adulterated samples. (Payn et al. 2025, Sadef et al. 2023)

A look at turmeric adulteration in various geographical areas shows clear differences. In regions where the regulations and their enforcement are relatively more strict, i.e., Australia, Canada, the European Union, Japan, New Zealand, South Korea, and the United States, spice adulteration appears to be less of an issue, while in other areas, e.g., Southern Asia or South America, the use of synthetic colorants and diluents such as corn starch still seems to be quite common. In the dietary/food supplement category, adulteration with synthetic curcumin is the most common issue. A question that remains unanswered is whether curcumin is the only synthetic curcuminoid used to adulterate turmeric extracts. The two studies (You et al. 2022, NOW FOODS 2021) using ^14^C isotope analysis to calculate the biobased content used the whole extract for their tests, so the exact identity of the material(s) derived from fossil sources was not determined. However, in one of these studies, a lower biobased content correlated with high (> 85.4%) relative curcumin content, suggesting that the synthetic material is solely curcumin. (You et al. 2022) On the other hand, Girme et al. (Girme et al. 2020) who used a byproduct of the curcumin synthesis as an indicator for the presence of synthetic curcumin/curcuminoids, noticed that three out of four extracts containing the byproduct had a “normal” ratio of curcumin:demethoxycurcumin:bisdemethoxycurcumin, usually 60-80% curcumin, 15-27% demeothoxycurcumin, and <5-15% bisdemethoxycurcumin. (Nelson et al. 2017, Perini et al. 2023) This prompted the authors to express doubts about the natural origin of all three of the main curcuminoids in the samples having a normal ratio of these constituents. Hence, it is possible that some marketed products contain synthetic demethoxycurcumin and bisdemethoxycurcumin in addition to synthetic curcumin. Other types of adulteration such as synthetic dyes or substitution with other *Curcuma* species are rare in turmeric supplements.

This scoping review has several limitations:

1. Using published data can provide only an estimate of adulteration at best as the information is heterogeneous,
2. Results from a limited number of samples may not represent the entire market,
3. Analytical methods may not have been validated for the specific analyte, and
4. Adulteration schemes frequently change over time. (Orhan et al. 2024)

However, large-scale investigations, such as the efforts by the European Commission’s Joint Research Center, can give a good idea about the prevalence of turmeric adulteration in Europe. On the other hand, for Oceania, Africa, and South America, the number of samples tested is very low. Hence, it is not possible to draw any conclusions about the quality and authenticity of the turmeric in these markets. In areas where the sample numbers are higher, data on adulteration may be skewed due to the narrow focus of the analytical test methods (e.g., testing only for lead chromate or metanil yellow) or due to the sampling process (e.g., in India, samples from the organized sector, which consists of licensed, registered, and formally regulated businesses, have lower adulteration rates than those from the unorganized sector, i.e., enterprises that lack formal contracts, social security, or government regulation. Therefore, the adulteration rate depends on the sector from which the samples were obtained).

As mentioned above, many (*n* = 19) of the selected publications did not use a validated analytical method, and in some cases, the method validation was unclear or could not be assessed. This may be in part because of a lack of harmonized guidance on validation of qualitative methods, although standard-setting organizations, such as the European Directorate for the Quality of Medicines & HealthCare (EDQM) and United States Pharmacopeial Convention, have published minimum requirements for identification and limit tests. (United States Pharmacopeia 2017, European Directorate for the Quality of Medicines and Healthcare 2020) These include an assessment of the specificity, and – for adulterants at low concentrations such as food dyes – at least the limit of detection.

When the assessment was restricted to papers with validated test methods, 133 of 949 turmeric samples were considered to be adulterated, which is an adulteration rate of 14.0%. Interestingly, the drop in adulteration percentage was due to a substantially lower adulteration rate of turmeric spice (57 of 586 samples, or 9.7%), whereas the adulteration rate for food and dietary supplements increased to 27.1% (62 of 229 samples). The remaining 134 samples could not be assigned to either of the two categories.

A lack of a formal validation is particularly noticeable with genetic test methods and in papers where turmeric adulteration was determined by wet chemistry methods. The genetic tests were exclusively used to detect bulking agents or other *Curcuma* species in powdered turmeric sold as spice. Therefore, some of the limitations seen with extracts, where DNA is often of low quality and/or degraded, do not apply. Three of the ten papers reporting on the use of DNA-based methods included quantitative data. (Oh and Jang 2020, Maquet et al. 2021, Vostrikova et al. 2021) In these cases, the authors were able to distinguish between intentional adulteration and accidental contamination at low concentrations.

Wet chemistry methods included, for example, a Lugol test to detect the presence of starch, (Payn et al. 2025, DE Sales Mélo et al. 2021) or the hydrochloric acid test to detect the presence of metanil yellow. (Payn et al. 2025, Verma et al. 2022) Such screening tests are often used in areas where more expensive analytical instruments are unavailable, and hence these tests may represent the best option based on the infrastructure at hand. Ideally, such tests should be confirmed in more specific assays, e.g., botanical microscopy for the detection of starch, or HPLC-UV/Vis methods for the food dyes.

The results raise questions about the impact on the benefits and safety of the adulterated turmeric products. A major concern with regard to the safety of turmeric spice is the presence of elevated lead levels due to the addition of undeclared lead chromate of other lead salts. Such practices have been directly linked to elevated blood lead levels in children in rural Bangladesh. (Forsyth et al. 2019a, Forsyth et al. 2019b) According to the World Health Organization (WHO), lead exposure can lead to numerous health issues, particularly impacting the development of the central nervous system in children. Lead also causes serious health problems in adults, including an increased risk of high blood pressure, heart disease and stroke, and kidney damage. In pregnant women, lead exposure can reduce fetal growth and lead to preterm birth. (World Health Organization 2024) Long-term exposure to chromate has been linked to respiratory conditions, ulcerations of the nasal septum, as well as lung and nasal cancer. (Calvo-Cerrada et al. 2021) Additionally, the azo dye Sudan I is considered genotoxic and carcinogenic. Due to the structural similarity with Sudan I, and data from *in vitro* and animal tests, other azo dyes that are used to adulterate turmeric, such as metanil yellow and Sudan II, Sudan III, and Sudan IV, are classified as potentially genotoxic and possibly carcinogenic, and therefore have been banned by many countries. (Anton et al. 2005, Khan et al. 2020, Nisa et al. 2016). Adulteration of turmeric dietary supplements with synthetic curcumin, which appears to be the most common authenticity issue observed with such products, has not been linked to any negative adverse effects, although analysis of 17 products associated with hepatotoxicity case reports in Italy showed that 11 of the products did not contain the demethoxylated curcuminoids and hence were made with synthetic curcumin. One byproduct of the curcumin synthesis, including (1*E*,4*Z*)-5-hydroxy-1-(4-hydroxy-3-methoxyphenyl) hexa-1,4-dien-3-one, and elevated concentrations of boron, were identified in products containing synthetic curcumin by Girme et al. (Girme et al. 2020) While boron is considered safe at the concentrations (below 500 mg/kg) found in the adulterated turmeric dietary supplement products, data of the safety of (1*E*,4*Z*)-5-hydroxy-1-(4-hydroxy-3-methoxyphenyl) hexa-1,4-dien-3-one are lacking.

The adulterants used as bulking agents, such as corn starch or maltodextrin, are not a safety risk. However, their presence will likely reduce the taste experience of turmeric spice, or the health benefits expected from turmeric food and dietary supplements.

## Conclusions

This scoping review provides an estimate of the adulteration rate for turmeric products (20.0%), and turmeric sold as spice (20.4%) and as dietary/food supplements (22.0%). There are substantial differences in the type of adulteration depending on the market channel. While undeclared synthetic dyes, diluents such as starches, paprika, cumin, or annatto are often used to adulterate turmeric spice, the sale of synthetic curcumin labeled as turmeric extracts is the most common type of adulteration for dietary/food supplements. There are also differences in adulteration depending on the geographic location. As an example, adulteration with lead chromate has been reported mainly from Southern Asia. Dilution with starches, and adulteration with other dyes, and red-colored spices is a global concern. Substitution of *C. longa* with other *Curcuma* species has been predominantly described in China, the Middle East, and Southern Asia. On the other hand, the use of synthetic curcumin in dietary/food supplements appears to be more common in Europe and North America.

Many analytical methods to detect adulteration exist, with liquid chromatography (either HPLC or UHPLC) using a UV/Vis or MS detector being the most commonly used. Due to the different types of adulterants, a combination of orthogonal laboratory analytical methods for authentication of commercial turmeric provides the best approach to detect adulteration, both in turmeric spice and in dietary/food supplements.

As exemplified by the reduction in lead chromate-adulterated turmeric powder in Bangladesh, (Forsyth et al. 2023) a combination of educational measures, increased testing by authorities, and stricter enforcement policies can have a positive impact on the quality and authenticity of turmeric products sold to consumers.

## Data Availability

The data that support the findings of this study are openly available at https://docs.google.com/spreadsheets/d/1ZFHr8l-e1_rwZ3S5ol7NumLYjxuZVklsfCfZR616Je8/edit?usp=sharing.
